# Elevated Graves’ Disease-Specific Thyroid-Stimulating Immunoglobulin and Thyroid Stimulating Hormone Receptor Antibody in a Patient With Subacute Thyroiditis

**DOI:** 10.7759/cureus.19448

**Published:** 2021-11-10

**Authors:** Anu Alvin Mathew, Roshin Papaly, Alvin Maliakal, Lakshya Chandra, Mc Anto Antony

**Affiliations:** 1 Endocrinology, Amala Institute of Medical Sciences, Thrissur, IND; 2 Endocrinology, University of Oklahoma College of Medicine, Oklahoma City, USA; 3 Internal Medicine, Southern Tennessee Internal Medicine, Lawrenceburg, USA; 4 Internal Medicine, Bon Secour St. Francis Hospital, Greenville, USA; 5 Endocrinology, Diabetes and Metabolism, Medical University of South Carolina, Anderson, USA

**Keywords:** graves´disease, hypothyroid, tsh receptor antibody, thyroid stimulating immunoglobulin tsi, subacute thyroiditis

## Abstract

Subacute thyroiditis can be rarely associated with autoimmune thyroid disorders. It includes Graves' disease which is characterized by the presence of a highly specific antibody known as thyroid-stimulating hormone (TSH) receptor antibody (TRAb). There are three types of TRAb: TSH receptor stimulating antibody (TSAb) which stimulates the TSH receptor causing Graves' disease, TSH receptor blocking antibody (TBAb) which blocks the TSH receptor causing hypothyroidism, and a neutralizing antibody which does not alter the thyroid function. There are two assays used to check the TRAb: the thyroid-stimulating immunoglobulin (TSI) assay and the TSH receptor-binding inhibitor immunoglobulin (TBII) assay out of which the TSI assay measures the stimulating antibody which is specific for graves' disease. Although autoimmune thyroid disorders can rarely occur following subacute thyroiditis, their clinical presentation is usually compatible with the type of antibody detected in the patient’s serum.

We present a unique case of a 44-year-old patient who presented with subacute thyroiditis followed by the development of persistent hypothyroidism even in the presence of elevated Graves' disease-specific TSI and TRAb.

## Introduction

Graves’ disease is the most common cause of hyperthyroidism in which antibodies directed against the thyroid-stimulating hormone (TSH) receptor cause continuous stimulation of the thyroid gland leading to hyperthyroidism. The antibody measured is known as TSH receptor antibody (TRAb) and there are two methods of measuring the TRAb which include the thyroid-stimulating immunoglobulin (TSI) assay and the TSH receptor-binding inhibitor immunoglobulin (TBII) assay. The TSI assay has a very high sensitivity (96%) and specificity (99%) for the diagnosis of Graves’ disease [1]. Subacute thyroiditis is a less common cause of hyperthyroidism and is presumed to be caused by a viral infection. It is typically characterized by hyperthyroidism followed by a transient hypothyroid phase with full recovery to the euthyroid state [2]. Very rarely, subacute thyroiditis is associated with autoimmune thyroid disorders [3,4]. We present an extremely unique case of a patient with persistent hypothyroidism who presented with subacute thyroiditis in the presence of significantly elevated Graves’ disease-specific antibodies.

## Case presentation

**Figure 1:**
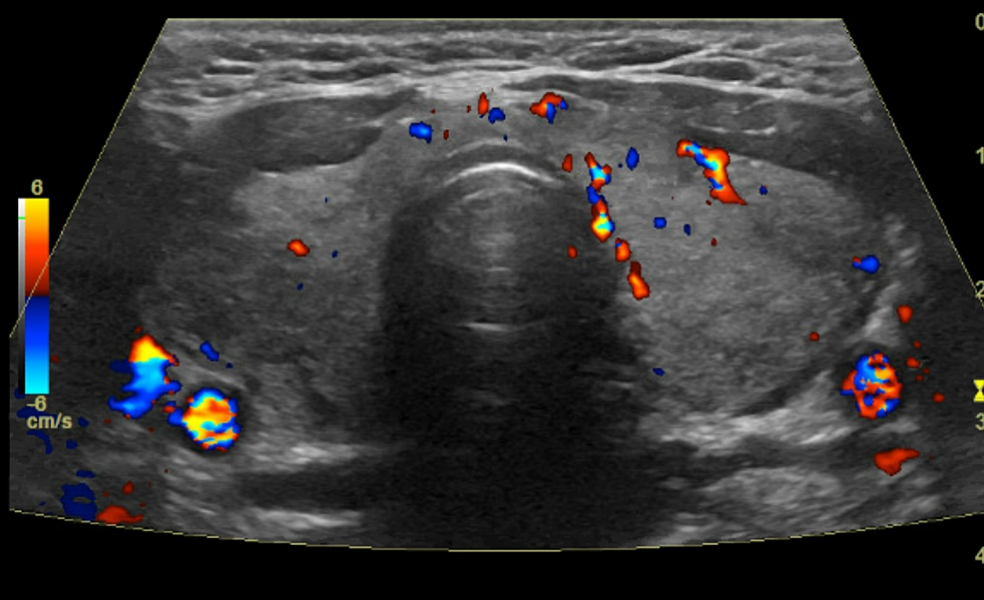
Thyroid ultrasound demonstrating heterogenous echotexture with diminished blood flow on color doppler

A 44-year-old female initially presented to her primary care physician in October 2018 with complaints of anterior neck pain, palpitations, increased sweating, and fatigue which started two weeks prior to presentation. On clinical examination, she had signs of thyrotoxicosis along with anterior neck swelling and tenderness. Laboratory evaluation confirmed hyperthyroidism with a suppressed TSH 0.022 mIU/L (0.4-4.5 mIU/L), elevated free thyroxine (FT4) 2.34 ng/dl (0.8-1.8 ng/dl), and elevated total thyronine (T3) 247 ng/dl (71-180 ng/dl). Erythrocyte sedimentation rate (ESR) was also elevated at 66 mm/hour (0-25 mm/hour). Thyroid ultrasound (Figure 1) showed diffusely heterogenous echotexture of bilateral thyroid lobes with diminished thyroid blood flow on color doppler. Also noted were a poorly defined 1.8 cm right mid to inferior pole nodule and a poorly defined 2.2 cm left mid pole nodule, which appeared to be pseudo-nodules. Due to a strong clinical suspicion for thyroiditis, the patient was treated with prednisone. Subsequently, the patient presented to the endocrinology clinic in April 2019 for the initial consultation. She reported experiencing a gradual improvement with the resolution of all the symptoms with the use of prednisone. However, she began to experience weight gain, fatigue, cold intolerance, and constipation after a few months of her initial presentation. The symptoms were highly suggestive of hypothyroidism, which was confirmed on subsequent laboratory evaluation with an elevated TSH 41 mIU/L and a low FT4 0.79 ng/dl. The TSI and TRAb were also checked along with the thyroid function tests due to the patient's history of initial presentation with hyperthyroidism. Interestingly, the TSI and TRAb were both significantly elevated at 63.1 IU/L (0-0.55 IU/L) and 68.5% (<16%), respectively, even though the patient was hypothyroid clinically and biochemically. A repeat thyroid ultrasound showed homogeneous thyroid gland echotexture with no discrete nodules. Levothyroxine therapy was initiated at a dose of 75 mcg/day in April 2019. During subsequent follow-ups, the levothyroxine dose had to be adjusted and the patient achieved a euthyroid state at a dose of 137 mcg/day and patient has remained on the same dose since October 2019. Due to the development of persistent hypothyroidism, the thyroid peroxidase (TPO) antibody was checked, which was in the normal range but the repeat TRAb was still elevated at 4.75 IU/L (<2.0 IU/L) in February 2020 which is 16 months since the initial presentation of the subacute thyroiditis. Table 1 gives a brief summary of these investigations.

**Table 1:** Summary of investigations TSH: thyroid-stimulating hormone, FT4: free thyroxine, TT3: total tri-iodothyronine, FT3: free thyronine, ESR: erythrocyte sedimentation rate, TRAb: TSH receptor antibody, TSI: thyroid-stimulating immunoglobulin, TPOAb: thyroid peroxidase antibody, NC: not checked

	Summary of investigations						
Labs (reference range)	10/2018	04/2019	07/2019	10/2019	02/2020	05/2020	12/2020
TSH (0.4-4.5 mIU/L)	0.022 mIU/L	41.0 mIU/L	16.55 mIU/L	11.27 mIU/L	1.05 mIU/L	1.70 mIU/L	1.52 mIU/L
FT4 (0.8-1.8 ng/dl)	2.34 ng/dl	0.79 ng/dl	1.0 ng/dl	1.0 ng/dl	1.2 ng/dl	1.3 ng/dl	NC
FT3 (2.3-4.2 pg/ml)	NC	NC	3.0 pg/ml	2.7 pg/ml	3.0 pg/ml	2.7 pg/ml	NC
TT3 (71-180 ng/dl)	247 ng/dl	NC	NC	NC	NC	NC	NC
ESR (0-25 mm/hr)	66 mm/hr	NC	NC	NC	NC	NC	NC
TRAb (<2.0 IU/L)	NC	NC	68.5% (<16%)	NC	4.75 IU/L	NC	NC
TSI (0.0-0.55 IU/L)	NC	63.1 IU/L	NC	NC	NC	NC	NC
TPOAb (<9IU/ml)	NC	NC	NC	NC	<1 IU/ml	NC	NC
Thyroid Ultrasound	Pseudo nodules	Normal	NC	NC	NC	NC	NC
Weight (lbs)	205	224	228	231	231	NC	234
Clinical presentation	Hyperthyroidism	Hypothyroidism	Hypothyroidism	Hypothyroidism	Euthyroid	Euthyroid	Euthyroid
Treatment course	Prednisone	Levothyroxine 75 mcg/day	Levothyroxine 100 mcg/day	Levothyroxine 137 mcg/day	Levothyroxine 137 mcg/day	Levothyroxine 137 mcg/day	Levothyroxine 137 mcg/day

## Discussion

TRAbs are of three types [5,6]: TSH receptor stimulating antibody (TSAb), TSH receptor blocking antibody (TBAb), and neutralizing antibody. Among the three sub-types, the clinically significant ones are the TSAb which stimulates the TSH receptor causing Graves’ disease, and the TBAb which blocks the TSH receptor causing hypothyroidism. There are two methods to check the TRAb; the TSI assay which measures the stimulating antibody and the TBII assay which measures the stimulating, blocking, and neutralizing antibodies. Hence, the TBII only indicates the inhibition of TSH-binding to the TSH receptor but cannot differentiate if TRAb is stimulatory or inhibitory. To know whether TRAb is stimulatory or inhibitory, we should individually measure the TSAb and TBAb [5,7]. But the prevalence and functional significance of TBAb in autoimmune hypothyroidism has been less well investigated compared to TSAb [8].

Sub-acute thyroiditis is an inflammatory condition of the thyroid which typically presents with tenderness and swelling of the thyroid gland during the hyperthyroid phase, followed by a transient hypothyroid phase with full recovery to the euthyroid state. However, permanent hypothyroidism can be seen in 15% of patients with subacute thyroiditis. During the initial stage of thyroiditis, all patients have laboratory evidence of hyperthyroidism which includes a low TSH level with an elevated free T4 and free T3 level. There is also an elevation in the ESR to levels above 50 mm/hour, suggestive of the underlying inflammatory process and the serum thyroglobulin (Tg) levels are elevated suggesting thyroid follicular cell damage. A radioactive iodine uptake (RAIU) test is useful in such cases as it helps to differentiate between endogenous hyperthyroidism which is typically associated with high 24-hour iodine uptake and thyroiditis which demonstrates a low or absent 24-hour iodine uptake. Our patient presented with anterior neck pain and tenderness, and an elevated ESR to suggest sub-acute thyroiditis as the etiology of hyperthyroidism. An RAIU test would have provided good supporting evidence for thyroiditis. However, given the typical clinical presentation for sub-acute thyroiditis, our patient was initiated on prednisone once the lab work confirmed the hyperthyroidism.

Although sub-acute thyroiditis is a self-limiting disease of viral etiology, it can be associated with autoimmune antibodies. The antibody that is detectable in Graves’ disease is known as TRAb and it has also been reported following thyroiditis. Iitaka et al. reviewed 1,697 patients with subacute thyroiditis and found 38 patients positive for TRAb out of which, the patients who were positive for TSAb developed hyperthyroidism and the patients with TBAb developed hypothyroidism [3]. However, our case is unique because our patient had persistently positive TRAb and TSI antibodies following sub-acute thyroiditis but remained persistently hypothyroid requiring initiation of thyroid hormone replacement.

Likewise, the antibodies that are detectable in Hashimoto’s thyroiditis are serum TPOAb and/or serum thyroglobulin antibody (TgAb), and both antibodies can be present at low titers in thyroiditis. Nishihara et al. studied TgAb and TPOAb in 40 patients in the early phase of subacute thyroiditis [4]. Patient samples were positive for either or both TgAb and TPOAb initially, but the titers decreased or disappeared afterward. In comparison, although our patient was clinically and biochemically hypothyroid, the TPOAb was negative which suggests a high possibility for the presence of TBAb, which is the blocking type of TRAb.

Autoimmune response to the damage of the thyroid follicular cells and immune complex formation is considered a major mechanism of the development of autoimmune thyroid antibodies in thyroiditis [9]. Bliddal et al. examined the patterns of thyroid antibodies and circulating immune complexes (CIC) during sub-acute thyroiditis in 10 patients. The TBII and CIC were positive in all patients and the changes in CIC levels paralleled the changes in TBII. Similar pathophysiology may have occurred in our patient who developed autoimmune thyroid antibodies as an immune response following thyroid follicular cell damage and the antibodies have persisted for a long time.

The presence of the TRAb or TSI antibodies could also be a predictor of Graves’ disease in the future [10]. Iitaka et al. described a patient who sequentially developed hypothyroidism positive for TBAb, followed by hyperthyroidism positive for TSAb, after an episode of subacute thyroiditis. However, our case is unique compared to the above case since our patient has remained persistently hypothyroid despite the persistent presence of TRAb and TSI antibodies which are highly sensitive and specific for graves’ disease. Nevertheless, close monitoring is required for our patient due to the possibility of developing Graves’ disease in the future.

Even though TRAb is persistently positive and the TPO was negative, the unexplained hypothyroidism in our patient is most likely due to the presence of TBAb which could not be tested due to the low prevalence and unavailability of the test in the U.S.

## Conclusions

Autoimmune thyroid disorders have been rarely reported following subacute thyroiditis and when they occur, their clinical presentation is usually compatible with the type of antibody detected in the patient’s serum, unlike our unique case in which the patient had a positive TSI antibody and a persistently positive TRAb but still developed persistent hypothyroidism instead of hyperthyroidism. Our case emphasizes the need for long-term monitoring due to the risk of developing Graves’ disease in the future. Our case also emphasizes the need to develop the TBAb bioassays to help understand the reason for such an unusual presentation.
